# Structural insights into N-terminal methionine cleavage by the human mitochondrial methionine aminopeptidase, MetAP1D

**DOI:** 10.1038/s41598-023-49332-6

**Published:** 2023-12-15

**Authors:** Yeon Lee, Hayoung Kim, Eunji Lee, Hyunggu Hahn, Yoonyoung Heo, Dong Man Jang, Kihyuck Kwak, Hyo Jung Kim, Hyoun Sook Kim

**Affiliations:** 1 Research Institute, National Cancer Center, Goyang, 10408 Republic of Korea; 2 Division of Medical Sciences, College of Medicine, Yonsei University, Seoul, 03722 Republic of Korea; 3 College of Pharmacy, Woosuk University, Wanju, 55338 Republic of Korea

**Keywords:** Enzyme mechanisms, Isoenzymes, Structural biology

## Abstract

Isozymes are enzymes that catalyze identical biological reactions, yet exhibit slight variations in structures and catalytic efficiency, which enables the precise adjustment of metabolism to fulfill the specific requirements of a particular tissue or stage of development. Methionine aminopeptidase (MetAP) isozymes function a critical role in cleaving N-terminal methionine from nascent proteins to generate functional proteins. In humans, two distinct MetAP types I and II have been identified, with type I further categorized into cytosolic (MetAP1) and mitochondrial (MetAP1D) variants. However, despite extensive structural studies on both bacterial and human cytosolic MetAPs, the structural information remains unavailable for human mitochondrial MetAP. This study was aimed to elucidate the high-resolution structures of human mitochondrial MetAP1D in its apo-, cobalt-, and methionine-bound states. Through a comprehensive analysis of the determined structures and a docking simulation model with mitochondrial substrate peptides, we present mechanistic insights into the cleavage process of the initiator methionine from mitochondrial proteins. Notably, despite the shared features at the active site between the cytosolic and mitochondrial MetAP type I isozymes, we identified distinct structural disparities within the active-site pocket primarily contributed by two specific loops that could play a role in accommodating specific substrates. These structural insights offer a basis for the further exploration of MetAP isozymes as critical players in cellular processes and potential therapeutic applications.

## Introduction

In biological systems, different enzymes that often catalyze the same reaction and exhibit functional redundancy are termed isozymes. Although they may facilitate the same reaction, they are not superfluous and typically operate at a distinct component level^1^. Further, they share a predominantly conserved structure; however, their substrate-like inhibitors are occasionally incompatible, as exemplified by human neuraminidase isozymes (hNEU1–4), where the antiviral drug zanamivir has potential inhibitory activity against hNEU2 and hNEU3 but not against hNEU1^2^. Hence, a comprehensive understanding of the distinct structural features and active-site residues unique to each isozyme can serve as a foundation for the tailored design of selective inhibitors.

Methionine aminopeptidase (MetAP) is a representative isozyme that catalyzes the excision of N-terminal methionine from newly synthesized proteins. Approximately 70% of proteins undergo the removal of N-terminal methionine, and this maturation process is crucial for protein stability, localization, and activation^3^. Particularly, in the context of the N-end rule, it has been elucidated that the N-termini of proteins starting with an *N*-alpha-acetylated Met or a free Met may serve as the site of *N*-ubiquitination by regulation of their steric shielding^4,5^, supporting the direct contribution of the N-terminal methionine excision to the protein stability. While most bacteria typically possess a single gene for MetAP, eukaryotes have isoforms of two types, type I and type II (in humans, these are hMetAP1 and hMetAP2, respectively). The latter is distinguished from the former by the insertion of approximately 60 residues into the catalytic domain, providing the distinct characteristics of MetAP type II^6–8^. hMetAP1 is implicated in cell cycle progression through the G2/M phase^9^, while hMetAP2 has gained significant attention owing to its identification as the physiological target of the anti-angiogenic natural products fumagillin and ovalicin^10^. Given that the absence (subclass a) or presence (subclass b) of N-terminal domain extensions serves as a distinguishing factor among MetAPs^7,11^, hMetAP1 and hMetAP2 can be classified as type Ib and IIb, respectively.

Unlike archaea and the cytoplasm of eukaryotes, where protein synthesis always begins with an unblocked NH_2_-methionine, the initial methionine is *N*-formylated in eubacteria and in the organelles such as mitochondria^12–14^. Therefore, in the case of human mitochondria, this necessitates the preceding deformylation process by peptide deformylases (PDFs) before the N-terminal methionine excision. In line with the identification of functional PDFs in human mitochondria^15,16^, an additional MetAP isoform, the organelle-targeted type Id MetAP1D (hMetAP1D), which is closely related to cytosolic type I hMetAP1 with a sequence identity of 46% in their catalytic domains but functions within the mitochondria, has been discovered in human cells^16^. While cytoplasmic hMetAP1 possesses a long N-terminal extension with a zinc finger domain involved in ribosome tagging through several exposed PXXP motifs for efficient target accessibility, hMetAP1D features a mitochondrial targeting signal at its N-terminal domain spanning residues 1–43, facilitating its localization to the mitochondria^16^. Phylogenetic analysis of human MetAPs^16,17^ suggests that cytosolic hMetAP1 likely originated from mitochondrial hMetAP1D via gene duplication and fusion to a ribosome-targeting domain. Recent studies have indicated that hMetAP1D is upregulated in colon cancer and that its suppression reduces the tumorigenic potential of colorectal cancer cells^17,18^. Moreover, it has been implicated in breast cancer incidence^19^. These findings suggest that hMetAP1D might serve an important role in oncogenesis. Notably, hMetAP1D exhibits the lowest efficiency in vitro in removing methionine from the Met-Ala-Ser substrate peptide compared to cytoplasmic hMetAP1 and hMetAP2^9,17^, suggesting the possibility of distinct substrate preferences and structural features specific to hMetAP1D. As the N-terminal processing becomes a target for drug development, a comprehensive understanding of human MetAPs involved in this mechanism is imperative. However, despite extensive structural studies on both bacterial and human cytosolic MetAPs^8,20–22^, as well as on human mitochondrial PDF^15^, the structural information remains unavailable for human mitochondrial MetAP.

We describe here the first crystal structures of human MetAP1D in the apo-, metal-, and methionine-bound states. By incorporating docking simulations with substrate peptides and comparing the structural characteristics of hMetAP1D to those of known MetAPs, our study provides a basis for designing novel selective anticancer agents.

## Results

### Crystal structures of hMetAP1D

**Figure 1:**
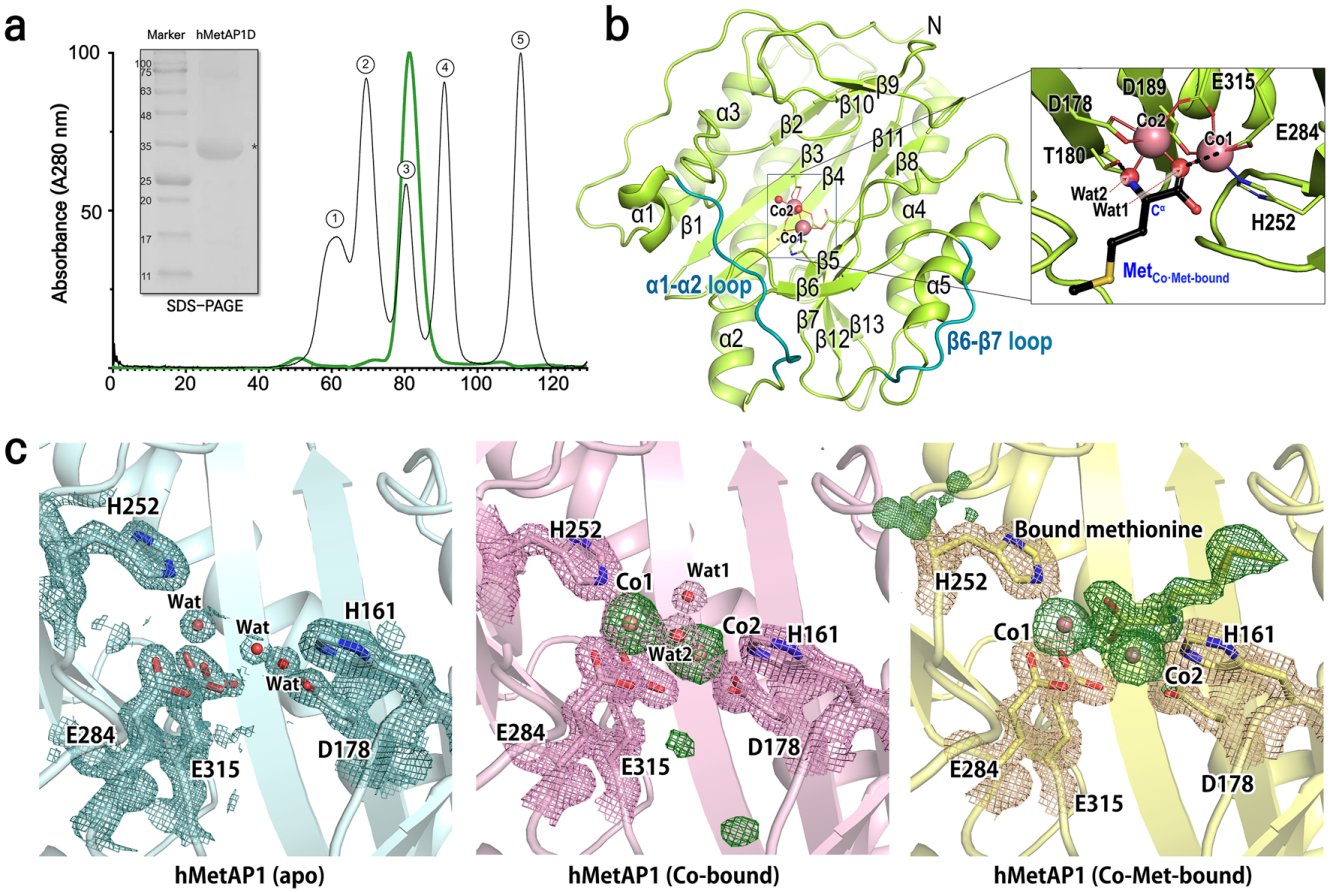
Overall structure of human MetAP1D. (**a**) Size exclusion chromatography results for the recombinant hMetAP1D proteins are shown, along with the corresponding SDS-PAGE. The major peak (green) corresponds to an expected molecular mass of approximately 34 kDa, with comparison to standard proteins: thyroglobulain (①, 670 kDa), bovine gamma-globulin (②, 158 kDa), chicken ovalbumin (③, 44 kDa), equine myoglobin (④, 17 kDa), and vitamin B12 (⑤, 1.35 kDa). (**b**) Ribbon diagram representing the overall structure of hMetAP1D in the metal-binding state (Co-bound structure). The bound cobalt ions and metal-bridging water molecules are denoted by spheres. The inset displays a closed-up view of the metal-binding ligand residues and the bound methionine from the superimposed Co-Met-bound structure, depicted as green and black sticks, respectively. (**c**) Electron density maps for the bound ligands and key residues of the hMetAP1D active site in the apo (blue), Co-bound (pink), and Co-Met-bound (yellow) states. The 2m*Fo-DFc* electron density maps for key active-site residues are contoured at a level of 1.0 σ with corresponding colors. The *mFo-DFc* omit maps for cobalt ions and the bound methionine, contoured at a level of 3.0 σ, are depicted in green.

To elucidate the precise structural basis underlying the initiator methionine removal process by MetAP1D within mitochondria, the crystal structures of human MetAP1D (hMetAP1D) encompassing the residues Arg44–Ala335 were determined at 1.39, 1.51, and 1.45 Å resolutions, for the metal-unbound (apo), dual cobalt ion-bound (Co-bound), and cobalt- and methionine-bound (Co-Met-bound) forms, respectively (Table 1). The N-terminal affinity tag and residues 44–48 were disordered in all three structures. Within the crystal lattice of hMetAP1D, wherein a monomer occupies the asymmetric unit, interactions between neighboring molecules predominantly involved loop contacts, chiefly involving terminal residues, without the formation of any discernible quaternary structure. This finding corroborates our size exclusion chromatography results (Fig. 1a), which showed that hMetAP1D exists as a monomer in solution.

**Table 1:** Data collection and refinement statistics for the hMetAP1D structures.

PDB Entry	8KHM apo	8KHN Co-bound	8KHO Co-Met-bound
Data collection			
Diffraction source	PLS-5C	PLS-5C	PLS-5C
Wavelength (Å)	0.97957	0.97957	0.97957
Temperature (K)	100	100	100
Space group	*C2*	*C2*	*C2*
a, b, c (Å)	77.4 81.2 48.9	78.9 81.0 49.0	79.4 81.1 49.0
α, β, γ (°)	90.0 102.3 90.0	90.0 102.2 90.0	90.0 102.3 90.0
Resolution range (Å)^a^	27.67–1.39 (1.44–1.39)	27.35–1.51 (1.57–1.51)	28.02–1.45 (1.50–1.45)
Total No. of reflections^a^	220,859 (22,356)	172,604 (18,324)	198,708 (19,819)
No. of unique reflections ^a^	58,426 (5720)	46,789 (4452)	53,296 (5335)
Completeness (%)^a^	99.2 (99.4)	98.9 (94.8)	99.3 (99.8)
Redundancy^a^	3.8 (3.8)	3.7 (3.9)	3.7 (3.7)
〈I/σ(I)〉^a^	12.83 (1.42)	8.61 (1.40)	10.40 (2.28)
*R*_sym_^a^	0.0620 (0.992)	0.0890 (1.022)	0.0710 (0.574)
*R*_meas_^a^	0.0730(1.151)	0.1044 (1.185)	0.0830 (0.669)
CC_1/2_^a^	0.999 (0.681)	0.997 (0.659)	0.997 (0.887)
Model refinement			
Overall B factor from Wilson plot (Å^2^)	14.12	17.33	16.10
No. of reflections, working set^a^	58,271 (5710)	46,566 (4451)	53,291 (5335)
No. of reflections, test set^a^	2958 (266)	2266 (221)	2673 (242)
Final *R*_work_^b^	0.190	0.193	0.183
Final *R*_free_^b^	0.209	0.214	0.209
No. of atoms/Average B factors (Å^2^)			
Protein	2262/18.54	2230/21.13	2,242/20.56
Methionine	–	–	9/20.38
Cobalt	–	2/17.06	2/14.42
PEG	10/39.10	10/44.60	10/43.36
Glycerol	12/34.39	–	–
Water	261/29.74	318/31.60	217/29.33
Root-mean-square deviations			
Bonds (Å)	0.007	0.007	0.007
Angles (°)	0.95	0.94	0.90
Ramachandran plot^c^			
Favored (%)	97.19	96.84	97.16
Allowed (%)	2.81	3.16	2.84
Rotamer^c^			
Favored (%)	96.83	97.17	96.79
Allowed (%)	1.13	1.33	0.89

^a^Values in parentheses refer to the highest resolution shell.

^b^*R*_work_ = Σ||*F*_obs_| −|*F*_calc_||/Σ|*F*_obs_|, where *R*_free_ is calculated for a randomly chosen 5% of reflections, which were not used for structure refinement and *R*_work_ is calculated for the remaining reflections.

^c^Values obtained using MolProbity.

**Figure 2:**
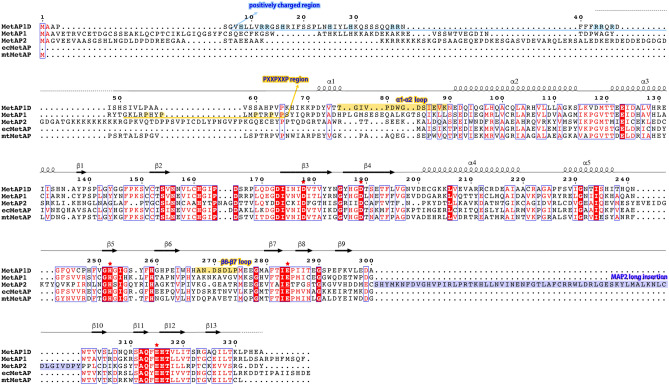
Structure-based sequence alignment for human and bacterial MetAPs. The structure-based sequence alignment for human and bacterial MetAPs was generated using the PROMALS3D program, integrating the sequences and structural information of human MetAP1D (hMetAP1D; UniProt ID: Q6UB82; PDB code: 8KHM), human MetAP1 (hMetAP1; UniProt: P53582; PDB: 2B3K), human MetAP2 (hMetAP2; UniProt: P50579; PDB: 1B6A), *Escherichia coli* MetAP (ecMetAP; UniProt: P0AE18; PDB: 1C21), and *Mycobacterium tuberculosis* MetAP (mtMetAP; UniProt: P9WK19; PDB: 3IU7). Secondary structural elements identified in the hMetAP1D crystal structure are indicated above the sequences, with the visible loop and invisible regions represented as solid and dotted lines in grey, respectively. Identical residues are depicted in white on a red background, whereas similar residues are highlighted in red on a white background. The blue box above the sequence indicates regions of conserved similarity. Metal-binding ligand residues are marked with red asterisks. Positively charged residues on the mitochondrial-targeting signal in the N-terminal domain of hMetAP1D, PXXP motif region of hMetAP1, and long insertion domain of hMetAP2 are highlighted in light blue, pale yellow, and light purple, respectively. The α1–α2 and β6–β7 loops of hMetAP1D are depicted with pale yellow backgrounds.

**Figure 3:**
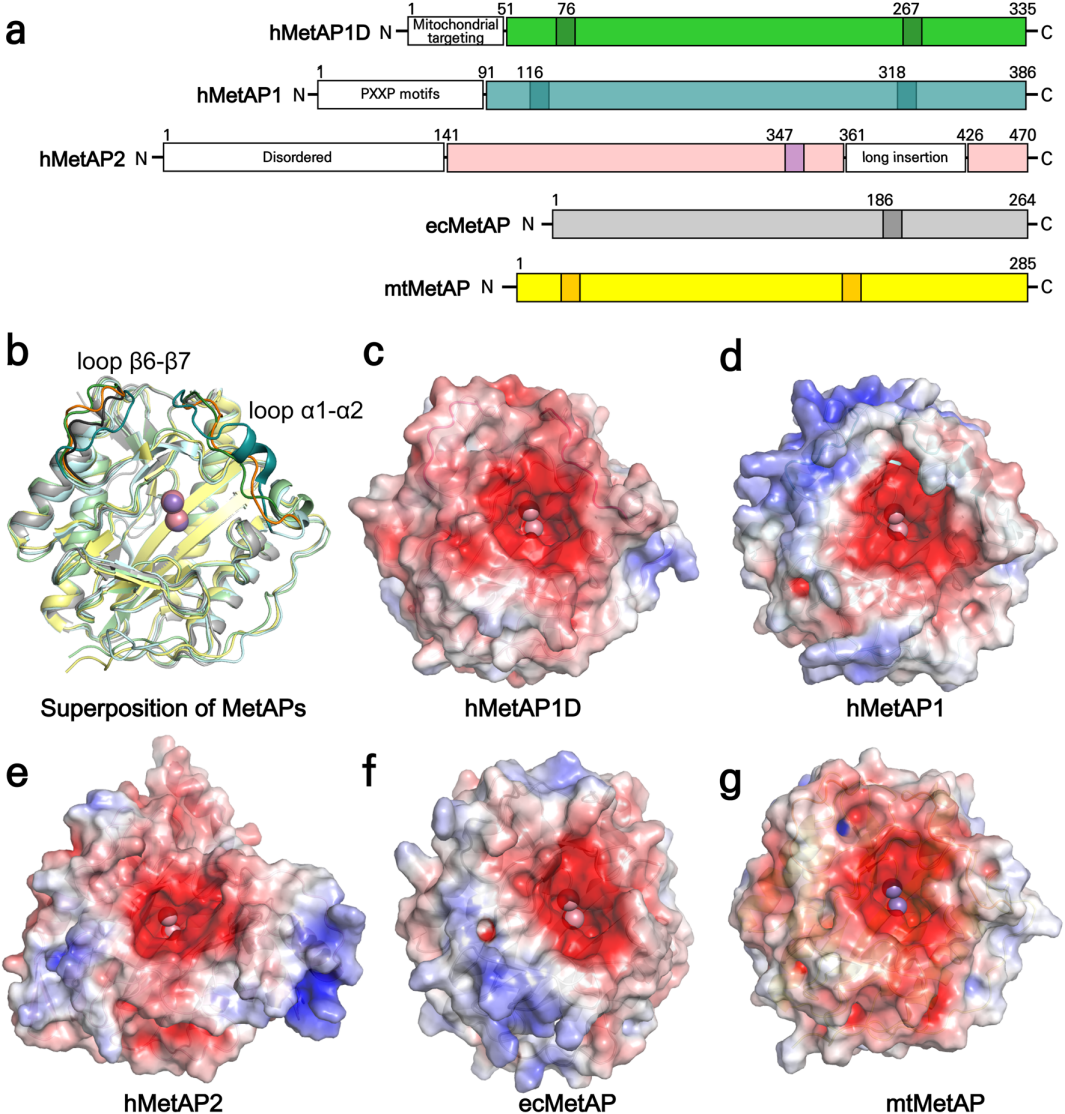
Structural summary of hMetAP1D and its homologs. (**a**) Domain architecture of human and bacterial MetAPs. The catalytic domains are highlighted by different colors, with the regions corresponding to the α1–α2 and β6–β7 loops of hMetAP1D indicated in relatively darker shades. (**b**) Cartoon representation of the superimposed structures of the catalytic domains of human and bacterial MetAPs, colored as in (**a**). (**c**–**g**) Electrostatic potential properties of the catalytic domains of hMetAP1D (**c**), hMetAP1 (**d**), hMetAP2 (**e**), ecMetAP (**f**), and mtMetAP (**g**) in the same view of the active site side, as calculated using APBS electrostatics plugins^45^ in PyMOL. Surfaces are colored according to their electrostatic potentials (scale bar -5.0 to + 5.0 kT/e; red, negative; blue, positive).

The overall structure of hMetAP1D adopts a characteristic architecture of typical MetAPs, resembling a pita bread fold and featuring long α-helices surrounding the exterior and aligned β-sheets comprising the inner pocket structure (Fig. 1b). The structure-based sequence alignment (Fig. 2) and DALI structural similarity searches^23^ indicated that hMetAP1D shares the overall fold and highly conserved key residues with the catalytic core domains of other human and bacterial MetAPs (Fig. 3), such as hMetAP1 [Protein Data Bank (PDB) code: 2B3K; Z-score of 43.8; sequence identity of 46.0%]^21^, hMetAP2 (PDB: 1B6A; Z-score of 24.4; sequence identity of 35.0%)^8^, and *Escherichia coli* MetAP (ecMetAP; PDB: 2MAT; Z-score of 38.1; sequence identity of 38.7%)^20^. Notably, the highest Z-score was obtained for *Mycobacterium tuberculosis* MetAP (mtMetAP; PDB: 3IU7; Z-score of 45.2; sequence identity of 40.8%)^24^. Antiparallel β-sheets primarily form a well-organized metal-binding active site for the enzymatic reaction, within which crystal structures of hMetAP1D revealed clear electron density corresponding to the binuclear Co^2+^ center or a methionine molecule (Fig. 1c). The Co-bound hMetAP1D structure, complexed with two cobalt ions (Co1 and Co2), exhibited metal-bridging water molecules (Wat1 and Wat2) and metal-binding ligand residues (Asp178, Asp189, His252, Glu284, and Glu315) (Fig. 1b), which are strictly conserved among other MetAPs (Fig. 2). Wat1, which bridges both Co ions, was shown to interact with Glu284, whereas Wat2, which binds only to Co2, formed a hydrogen bond with Thr180 (inset of Fig. 1b).

Comparison of the Co-bound structure with the apo- and Co-Met-bound structures revealed that their overall structures were nearly identical, with a root-mean-square (r.m.s.) deviation of 1.1 Å over 287 Cα atoms. No significant structural changes were detected in the active site upon the binding of metal ions or methionine. However, the subtle movements of water molecules and the side chains of several key amino acids were identified. In the apo structure, three water molecules, corresponding to the positions of Co1, Co2, and Wat2 shown in the Co-bound structure, were anchored by metal-binding ligand residues (left of Fig. 1c). Notably, Asp178 and Glu315 showed double occupancy, and the bound water molecules displayed relatively broad electron density maps, suggesting a less coordinated state. In the Co-bound structure, following the addition of two cobalt ions, the two acidic residues that had double occupancy in the apo state became stably coordinated with lower B-factor values (middle of Fig. 1c), thus establishing a robust interaction network between the metals and ligands (inset of Fig. 1b). When methionine (L-Met) was introduced, its main-chain amino group and one oxygen atom of the carboxyl group replaced the positions of Wat2 and Wat1 in the Co-Met-bound structure, respectively, illustrating the product-bound state (right of Fig. 1c), which is discussed in greater detail subsequently.

### Comparisons of hMetAP1D with other MetAPs

The structure-based sequence alignment, coupled with the superimposition of structures among hMetAP1D (type Id), hMetAP1 (type Ib), hMetAP2 (type IIb), ecMetAP (type Ia), and mtMetAP (type Ic)^14^, demonstrated that in the absence of the N-terminal mitochondrial targeting signal, the hMetAP1D structure retained the pita-bread fold and metal-binding residues (Figs. 2 and 3b). Despite their shared fold and sequence similarities, these were primarily confined to their catalytic domains due to the distinct domain organizations that define their respective subclasses. Notably, the hMetAP2 structure exhibited the type II-characteristic elongated insertion within the β9–β10 loop, which was absent from the structures of other type I MetAPs, including MetAP1D (Figs. 2 and 3a). Moreover, MetAP1D was shown to feature an additional N-terminal domain analogous to those of hMetAP1 and hMetAP2; however, this domain was characterized by an alternating pattern of hydrophobic and positively charged basic residues. Consequently, the N-terminal domain of hMetAP1D is considered to function in mitochondrial targeting^16^, rather than in ribosome attachment. The latter is typically orchestrated by a ribosome-targeting N-terminal motif, PXXPXXP, which was conspicuously absent in MetAP1D (Fig. 2). When superimposing the three-dimensional structures of different MetAPs, a uniform negative charge distribution within the active-site pocket was evident (Fig. 3c–g). However, the external surfaces of these structures displayed pronounced divergent electrostatic properties. It is pertinent to emphasize that hMetAP1D presented a more marked negative charge relative to those of other structures not only on the external surface of the active-site pocket but also across its entire external region (Fig. 3c). This accentuates its lower isoelectric point (pI), with a theoretical value of 5.68, juxtaposed with a pI value of 6.23 for hMetAP1. This observation is particularly noteworthy, given that proteins transported to the mitochondria typically possess high pI, supplemented by abundant hydrophobic patches^25^.

**Figure 4:**
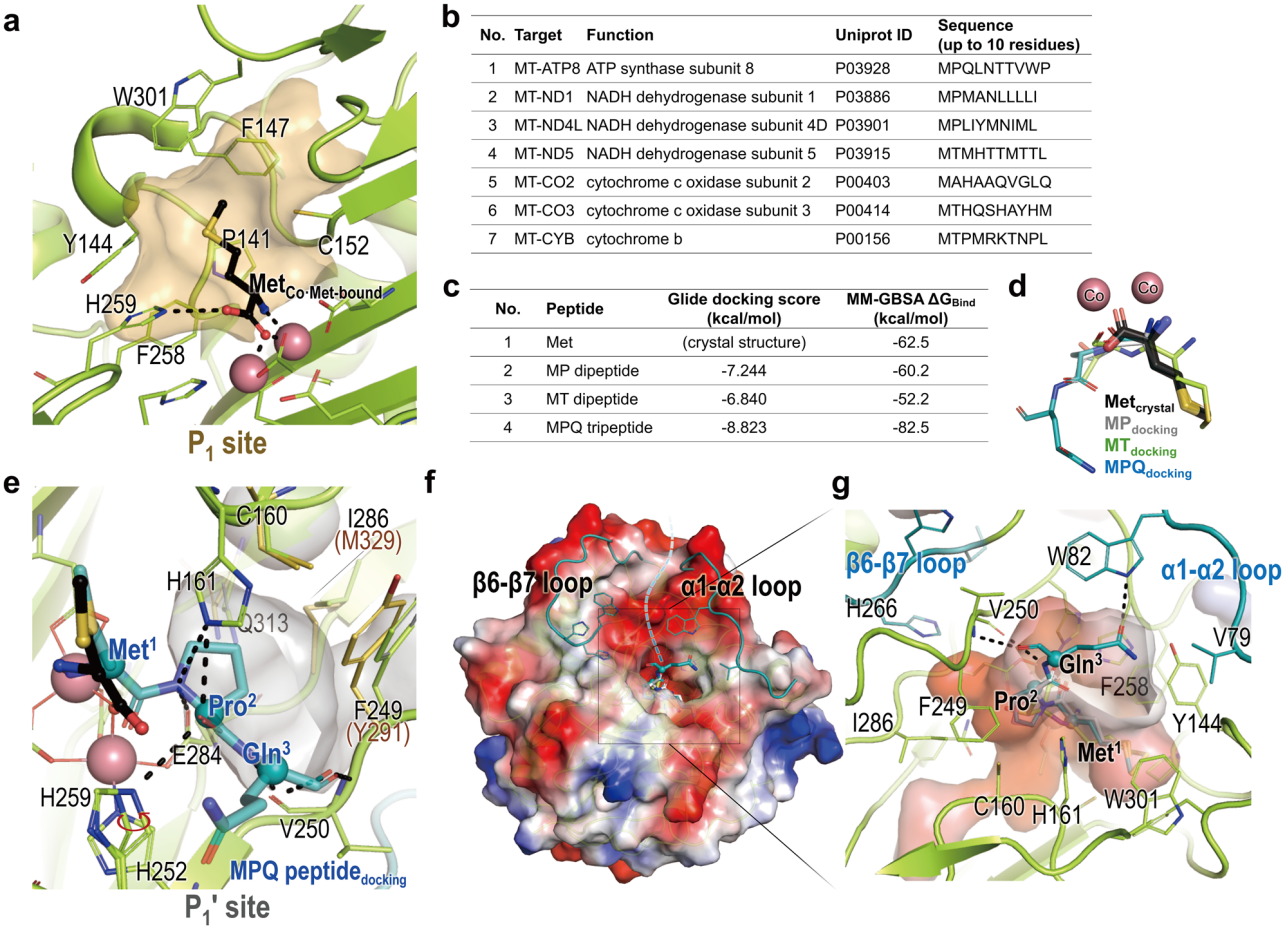
Substrate recognition by hMetAP1D. (**a**) Surface representation of the site of hMetAP1D that recognizes the initiator methionine (P_1_ subsite) in the Co-Met-bound structure, highlighted in orange. The residues constituting the subsite and the complexed methionine are depicted as green and black sticks, respectively. The dotted line denotes the hydrogen bond interaction of His259 with the oxygen atom of the carboxylate group of the bound methionine. (**b**) Seven mitochondrial proteins proposed as potential substrates of hMetAP1D^16^, with their respective N-terminal residues considered for the docking simulations. (**c**) Outcomes from molecular docking and MMGBSA binding free energy calculation with three substrate peptide variations. The binding free energy for the methionine bound in the Co-Met-bound structure was also calculated for comparison. (**d**) Stick representations of three substrate peptides and the product methionine after superimposing the docking model and the Co-Met-bound structures, with the cobalt ions of the Co-Met-bound structure depicted as spheres. (**e**) Surface representation of the P_1_′ subsite of hMetAP1D in the Met-Pro-Gln tripeptide (MPQ peptide)-docking structure model, colored in grey. The tripeptide and its surrounding residues are represented with teal and green sticks, respectively. For comparison, the residues of hMetAP1 constituting the P_1_′ subsite are shown in yellow-orange sticks. The methionine and the His259 residue from the superimposed Co-Met-bound structure are also shown in black and green sticks, respectively, with the red arrow denoting a conformational change of the side chain of His259 upon substrate peptide docking. Black dotted lines represent interactions between the tripeptide and hMetAP1D active-site residues. (**f**-**g**) Exterior (**f**) and pocket-only (**g**) views of the electrostatic potential surface of the profound active-site pocket of hMetAP1D, with the tripeptide stick model from the docking simulations. The α1–α2 and β6–β7 loops are emphasized, appearing both as surface features and in the teal-colored cartoon representation (**f**), while the characteristic residues surrounding the deep active-site pocket are depicted in green or teal sticks (**g**).

Furthermore, structural comparisons between these proteins revealed subtle yet significant disparities in their substrate recognition sites. While the Co-Met-bound structure of hMetAP1D facilitated the identification of the site that recognizes the initiator methionine (P_1_ subsite) (Fig. 4a), it exhibited similarities with the sites of hMetAP1 and bacterial MetAPs but slightly diverged from that of hMetAP2 (Table 2). Despite minor variations in the residues constituting the P_1_ subsite, their surface shape and electrostatic distribution of the subsite remains conserved, which is pivotal for recognizing the initiator methionine as the invariant residue in their enzymatic reaction. The P_1_ subsite within the hMetAP1D structure featured an array of bulky and hydrophobic residues on α1 and loops. Specifically, the residues Tyr144, Phe147, Phe258, and Trp301 were present, potentially aiding in the stabilization of the aliphatic side chain of the initiator methionine.

**Table 2:** Residues contributing to the P_1_ and P_1_′ subsites of MetAPs.

Subsite	hMetAP1D (this study)	hMetAP1^21^ (PDB: 2B3K)	hMetAP2^44^ (PDB: 1KQ9)	ecMetAP^20^ (PDB: 2MAT)	mtMetAP^24^ (PDB: 3IU7)
P_1_	Pro141	Pro183	Phe219	Cys59	Thr94
	Tyr144	Tyr186	Pro220	Tyr62	Tyr97
	Phe147	Phe189	Gly222	Tyr65	Phe100
	Cys152	Cys194	His382	Cys70	Cys105
	Phe258	Phe300	Met384	Phe177	Phe211
	Trp301	Trp344	Ala414	Trp221	Trp255
			Tyr444		
P_1_′	Cys160	Cys202	Ala230	Cys78	Cys113
	Phe249	Tyr291	Leu328	Tyr168	Phe202
	Glu284	Glu327	Glu364	Glu204	Glu238
	Ile286	Met329	Phe366	Met206	Met240
	Gln313	Gln356	Leu447	Gln233	Gln267
			Gln457		

Given the necessity for MetAPs to accommodate amino acids with small side chains as the second residue following the initiator methionine^26^, we aimed to identify the hMetAP1D residues (P_1_′ subsite) responsible for recognizing the second residue and the scissile bond between the first and second residues of substrate proteins via docking simulation. To elucidate the interaction between hMetAP1D and the N-terminus of substrate polypeptides, we performed computational docking of the dipeptide (Met-Pro or Met-Thr) or tripeptide (Met-Pro-Gln) derived from the N-terminal regions of seven mitochondrial proteins earmarked for recognition^16^ (Fig. 4b) against the active site of the Co-Met-bound hMetAP1D structure, where waters and the bound methionine have been removed. The docking outcomes of the premier pose, characterized by the lowest Glide docking score and Molecular Mechanics Generalized-Born surface area (MMGBSA) binding free energy (Fig. 4c), indicated that the positions and conformations of the initiator methionine residue from the three peptide variants were closely aligned with the bound methionine in the crystal structure of Co-Met-bound hMetAP1D (Fig. 4d,e). Within the tripeptide-docked structure, the path leading to the hMetAP1D active site was discerned, which was capable of accommodating N-terminal segments of up to three residues in length (Fig. 4f,g), with limited space available for the second residue. This observation underscores hMetAP1D’s preference for recognizing relatively small and uncharged amino acids such as proline, threonine, and alanine at the P_1_′ subsite, as depicted in the surface representation in Fig. 4e. While the residues comprising the P_1_′ subsite of hMetAP1D are largely conserved among MetAPs, the presence of the hMetAP1D-specific Ile286 residue, corresponding to Met329 of hMetAP1 and Phe366 of hMetAP2, appeared to afford slightly more space within the subsite, compared with the corresponding residues of other human MetAPs (Table 2 and Fig. 4e). Notably, the docking score and free binding energy (Fig. 4c) suggests that the Met-Pro dipeptide docks into the hMetAP1D pocket with greater stability than the Met-Thr dipeptide, highlighting its preference for the second-site residue.

### Comparisons of mitochondrial and cytosolic type I human MetAPs

**Figure 5:**
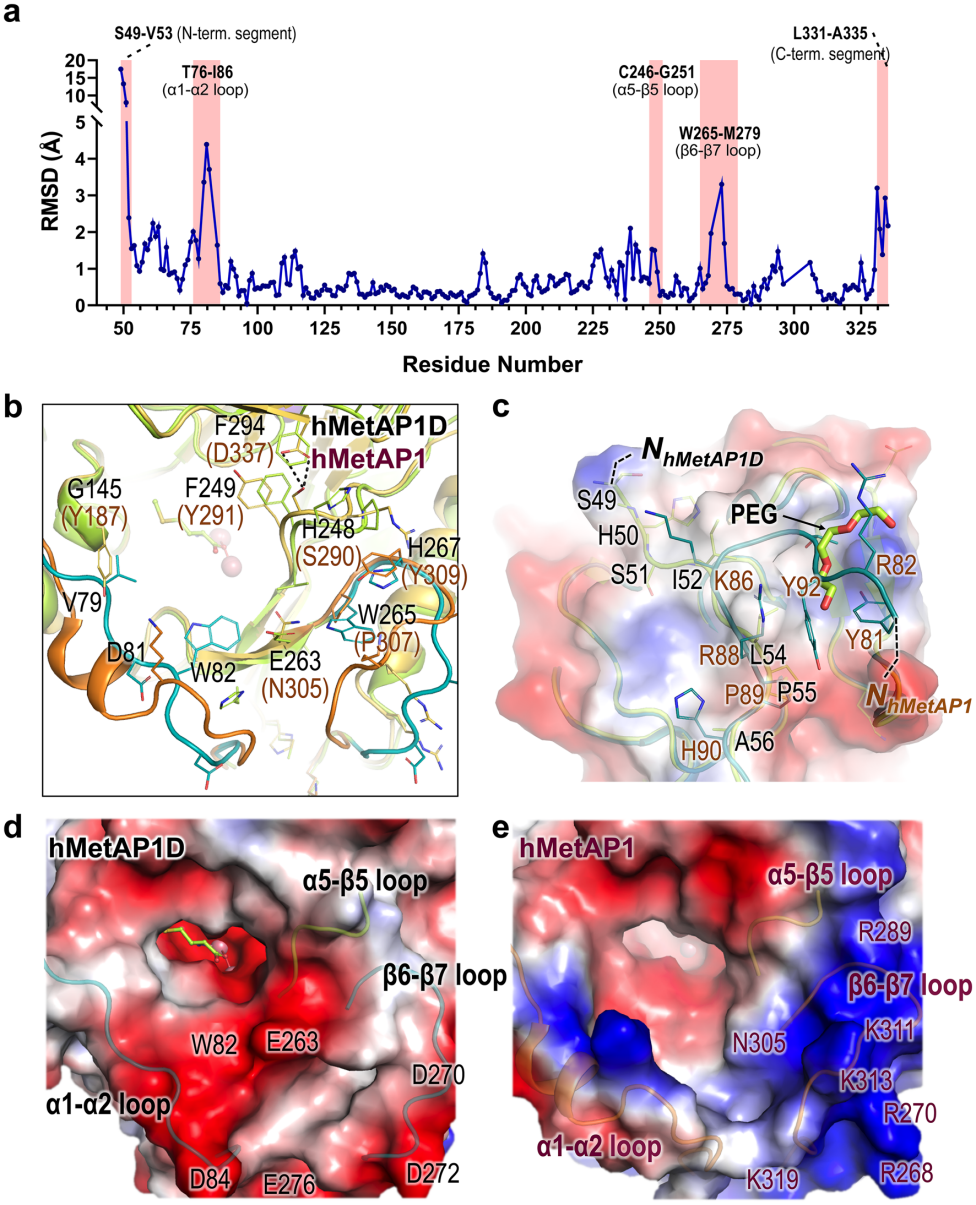
Structural comparisons of hMetAP1D and hMetAP1. (**a**) A plot of the Cα r.m.s. differences between hMetAP1D and hMetAP1 structures following secondary structure-based superimposition. Regions with significant r.m.s. differences are highlighted with pink background boxes. (**b**, **d**, **e**) Cartoons (**b**) and electrostatic potential surfaces (**d** and **e** for hMetAP1D and hMetAP1, respectively) of the entrance groove leading to the deep active-site pocket in their superimposed structures, seen from the same perspective. The α1–α2 and β6–β7 loops and the residues forming the groove are displayed in ribbon and stick models, respectively, representing hMetAP1D in green and hMetAP1 in orange. (**c**) Structural alignment of their N-terminal segments, represented in ribbon and surface views. Their N-termini are denoted with dotted black lines, and the polyethylene glycol molecule or N-terminal residues are illustrated in sticks.

Docking results for the Met-Pro-Gln tripeptide provided insights into the orientation of the main-chain carbonyl group at the third residue. This orientation was directed towards the groove established by the α1–α2 and β6–β7 loops (Fig. 4f), suggesting a potential pathway for accommodating consecutive amino acids beyond the third residue. These loops, distinguishing hMetAP1D from hMetAP1 and other MetAPs (Figs. 2 and 3), displayed significant r.m.s. differences between the mitochondrial hMetAP1D structure and the cytosolic hMetAP1 structure (Fig. 5a). Notably, the α1–α2 loop in the hMetAP1 structure included an additional α-helix, while hMetAP1D adopted a completely different loop conformation. Moreover, the bulky side chain of Tyr187 in hMetAP1, equivalent to Gly145 of hMetAP1D, replaced the position of Val79 on the α1–α2 loop of hMetAP1D, leading to an outward protrusion (Fig. 5b). The β6–β7 loop of hMetAP1D, which includes the characteristic Trp265 residue corresponding to Pro307 of hMetAP1, assumed a distinct conformation that extended outwardly (Fig. 5b). These variations in loop configurations and distinctive residues influenced the shape and size of the entrance tunnel into the profound pocket, culminating in the emergence of a shallower but more expansive entrance to the deep active-site pocket of hMetAP1D (Fig. 5d) relative to hMetAP1 (Fig. 5e). Notably, the Trp82 residue on the α1–α2 loop of hMetAP1D showed a crucial function in defining the entrance to the active-site pocket (Fig. 5d). Furthermore, the surface electrostatics around the groove of hMetAP1D exhibited a predominantly negative charge owing to the abundance of acidic residues, such as Asp84, Glu276, Asp270, Asp272, and Glu263 (Fig. 5d), in contrast to the positively charged residues that delineated the groove of hMetAP1 (Fig. 5e). In examining the protein structures, minor conformational variations were discernible in the α5–β5 loop vicinity near the deep pocket (Fig. 5b,d,e), indicating a subtle r.m.s. difference (Fig. 5a). This is likely attributed to the hydrogen bonds formed by Ser290 on the loop of hMetAP1 with Asp337, whereas the corresponding His248 and Phe294 residues of hMetAP1D establish π-π stacking interactions with Phe249 (Fig. 5b). These distinctive structural features surrounding the active-site pocket may confer the specificity required for human MetAP isozymes to adjust the cleavage efficiencies for their respective substrates.

In addition, the structures of hMetAP1D and hMetAP1 showed altered conformations in either the N- or C-terminal regions, characterized by considerable r.m.s. differences (Fig. 5a). When juxtaposed with the N-terminal segment (residues Tyr81–Pro100) containing PXXP motifs (^89^PHYPLMPTRP^98^) in the hMetAP1 structure, the N-terminal section (residues Ser49–Pro65) of hMetAP1D was notably devoid of the PXXP motif but demonstrated structural alignment in the Pro55–Pro65 region (Fig. 5c). Nonetheless, the Ser49–Leu54 loop region of hMetAP1D, as clearly elucidated by the observed electron densities, deviated in orientation from that of hMetAP1, forming multiple hydrogen bonds via His50 and Ser51 with Arg167, Gln170, and Asn308 (Fig. 5c). Notably, all three of our hMetAP1D structures revealed the presence of a polyethylene glycol (PEG) molecule on the path typically occupied by the N-terminal segment of hMetAP1 (Fig. 5c). These observations underscore the unique orientation and composition of the N-terminal segments of the two human type I MetAP isozymes that influence their respective functions.

### Proposed reaction mechanism of hMetAP1D

**Figure 6:**
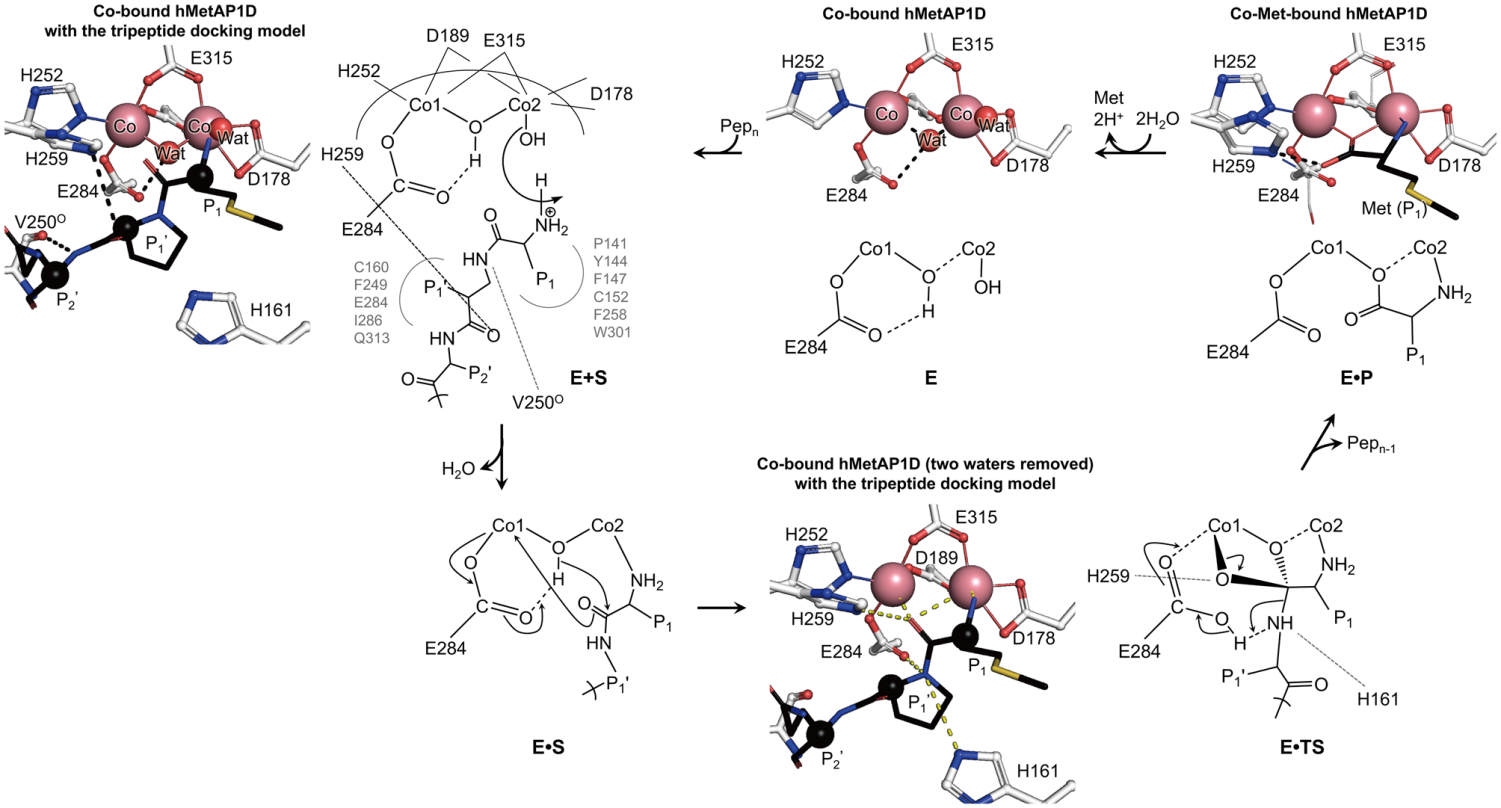
Proposed binuclear reaction mechanism for hMetAP1D. Schematic illustrations of the proposed catalytic mechanism of hMetAP1D, adapted and modified from previous studies^7,20^, with stick representations of the structural models for the respective states obtained from this study. For the E + S model, the binuclear center shown in the Co-bound structure is represented by the key residues in white sticks and metal spheres, superimposed with the tripeptide substrate in black sticks obtained from the docking simulations. These configurations were also used for the E•TS model after removing two metal-bridging water molecules from the Co-bound structure, illustrating potential interactions (yellow dashed lines) between the binuclear center and the tripeptide substrate in a transient state. The Co-Met-bound and Co-bound structures were used to depict the product-bound state (E•P) and enzyme-only state (E), respectively.

By incorporating the previously proposed binuclear catalytic mechanism for ecMetAP^20^, snapshots derived from high-resolution crystal structures representing the apo-, metal-, and product-bound states, as well as docking results with substrate peptides, collectively contributed to the establishment of a model elucidating the catalytic mechanism of hMetAP1D (Fig. 6). For the cleavage process, the N-terminal segment of substrate polypeptides accesses the active-site pocket via the groove formed primarily by the α1–α2 and β6–β7 loops, along with acidic charged residues such as Asp84, Glu263, and Asp272 (Fig. 5d). Upon the anchoring of the N-terminal residues of a substrate polypeptide (approximately three residues in length) in the active-site pocket bound with two cobalt ions (Fig. 4g), the initiator methionine and scissile peptide bond is precisely positioned through the coordination of Co2 and the backbone nitrogen atom, displacing the Co2-bridging water (or hydroxide) molecule (Wat2) (the E + S state of Fig. 6). This aligns with the structure of hMetAP1D docked with the tripeptide, where the main-chain amino group of the initiator methionine replaces the position of Wat2 shown in the Co-bound structure, and the scissile-bond carbonyl group closely approaches the water molecule (Wat1), bridging both cobalt ions. Activated Wat1 functions as a nucleophile, attacking the carbonyl moiety of the scissile peptide bond (E•S), and generating a proton that is transferred to Glu284. Our docking model with the tripeptide substrate illustrates that the side chain of Glu284 was likely to interact with the scissile amide bond probably for further proton transfer to the departing nitrogen atom (Figs. 4e and 6), providing a glimpse into a transition state (E•TS). The transient enzyme–substrate complex could be further stabilized by His161 and His259, as shown in the tripeptide-docked hMetAP1D model, where the slightly flipped side chain of His259, relative to its conformation in the Co-Met-bound structure (Fig. 4e), establishes hydrogen bonding interactions with the scissile-bond carbonyl group, together with the side chain of His161 (depicted as E•TS in Fig. 6). Upon detachment of the cleaved polypeptide from the N-terminal methionine, the produced L-methionine is bound to two metals (E•P), as clearly observed in the Co-Met-bound structure (Figs. 4a and 6), where His259 interacts with one oxygen atom of the carboxyl group of L-methionine. The release of the product from the two cobalt ions enables the insertion of a nucleophilic water molecule, preparing for the next reaction of hMetAP1D.

## Discussion

MetAP enzymes are a class of evolutionarily conserved metalloproteases responsible for cleaving the starter NH_2_-terminal methionine residue from nascent polypeptide chains. Although type I MetAPs are prevalent in eubacteria and type II in archaea, both types coexist in eukaryotes. The critical role of N-terminal methionine removal is evident from the lethal consequences of deleting all MetAP-encoding genes in eubacteria^27,28^ and yeast^29^. In mammalian systems, the functional significance of MetAPs has been primarily elucidated using MetAP inhibitors, including fumagillin, TNP-470, and ovalicin, which are known for their specificity toward hMetAP2, suggesting the involvement of hMetAP2 in tumor angiogenesis and metastasis^30–33^. Given the emerging significance of N-terminal processing as a potential target for drug development, it is essential to attain a comprehensive understanding of human MetAPs involved in this mechanism.

In this study, we present the first structure of human mitochondrial MetAP1D, which is responsible for mitochondrial N-terminal methionine excision and shares a pita-bread folded structure with cytoplasmic hMetAP1 and hMetAP2, albeit with weak sequence similarity to hMetAP2. Comparative structural analysis, including comparison with bacterial MetAPs, indicated that the catalytic domain of hMetAP1D exhibited significant structural similarities with type I MetAPs, such as hMetAP1, ecMetAP, and mtMetAP, confirming its classification within the type I subclass. Notably, the hMetAP1D structure exhibited distinct characteristics, particularly its prominently negative charge not only on the external surface of the active-site pocket but also across its entire external region, distinguishing it from the other MetAPs (Fig. 3). Moreover, the N-terminal segment of hMetAP1D, comprising residues Ser49–Leu54, modeled with clear electron densities, demonstrated a unique orientation, distinct from that of the corresponding N-terminal region of hMetAP1 containing the PXXP motifs. This observation suggests that while hMetAP1D shares an N-terminal domain with hMetAP1, it adopts a different conformation and composition in this region, likely contributing to its mitochondrial targeting function^16^ (Figs. 2 and 5c).

The crystal structures of hMetAP1D in apo-, cobalt-, and L-Met (product)-bound states were used to elucidate the structural features of each catalytic reaction step. Furthermore, we aimed to provide structural insights into the substrate-recognizing regions through docking simulations with substrate peptides. Leveraging the conformation of the bound methionine backbone amide in the Co-Met-bound structure, we performed docking studies using substrate polypeptides with two or three residues commonly found in the N-terminal regions of seven mitochondrial proteins. The docking results indicated that about three residues from the polypeptide substrate were appropriately positioned within the deep active-site pocket. Through a comprehensive approach, we gained insights into not only the P_1_ and P_1_′ subsites for substrate binding but also the recognition of the scissile peptide bond by hMetAP1D. These findings corroborate in-depth insights into the catalytic mechanism of hMetAP1D, which has been proposed by previous studies for ecMetAP^20^. Notably, the hMetAP1D-specific Ile286 residue, which comprises the P_1_′ subsite, was distinct from Met338 of hMetAP1 and Phe366 of hMetAP2, revealing a unique subsite environment that may influence its substrate preference.

Furthermore, our analysis unveiled structural features unique to hMetAP1D in the α1–α2 and β6–β7 loops, which comprise the entrance path leading to the active site. These loop regions, which assume significantly different conformations from those of the corresponding regions of hMetAP1, are characterized by abundant acidic residues and the Trp82 residue strategically positioned in front of the deep pocket. This arrangement contributes to the formation of an entrance groove leading to a profound active-site pocket, which serves as a pathway for accommodating consecutive amino acids beyond the third residue, as demonstrated in the tripeptide-docking model. We propose that these loops function akin to forceps, aiding in the proper assessment and docking of substrate polypeptides. Additionally, the higher distribution of negative charge on the surface of the entrance groove compared to that seen for hMetAP1 suggests that the negative charge near the active site may serve to attract the positively charged N-terminal signaling sequences of the mitochondrial substrate proteins^34^ (Figs. 2c and 4f).

In summary, our study provides valuable structural insights into the substrate and product recognition and catalytic mechanisms of hMetAP1D. In particular, the presence of a relatively negatively charged surface of hMetAP1D, coupled with considerable structural distinctions in the adjacent groove near its active site relative to the structure of cytoplasmic hMetAP1, underscores its unique functional properties. Although hMetAP1 and hMetAP1D share several critical features of the binuclear active-site center, variations in the shape of the pathway leading to the active site, influenced by specific loops and charge distributions, likely contribute to their context-dependent substrate selectivity. Collectively, our high-resolution structures of apo-, Co-, and Co-Met-bound states, in conjunction with substrate-docked models, provide intricate insights into the cleavage process. This comprehensive understanding could potentially guide the intelligent design of novel inhibitors targeting human MetAP proteins, serving as a foundation for future drug development.

## Methods

### Cloning, expression, and purification of recombinant hMetAP1D proteins

The full-length human MetAP1D gene (hMetAP1D; UniProt code: Q6UB28) was chemically synthesized (Bionics, Korea) and optimized for expression in *Escherichia coli*. The N-terminal-truncated human *MetAP1D* (44–335) was amplified by PCR and cloned into the pET-28a ( +) vector (Novagen, USA) using *NdeI*/*XhoI* restriction enzymes. The resulting recombinant hMetAP1D was fused to an N-terminal His_6_-tag. The cloned plasmid containing the hMetAP1D construct was transformed into BL21-CodonPlus(DE3)-RIPL *E. coli* for protein expression. The transformed cells were grown at 37 °C in Luria broth medium to an OD_600_ of 0.8, and the hMetAP1D recombinant protein expression was induced with 0.5 mM isopropyl 1-thio-β-D-galactopyranoside at 18 °C for 22 h. The harvested cells were resuspended in a lysis buffer [20 mM Tris–HCl (pH 7.9), 500 mM sodium chloride, 5 mM imidazole, 10% (*w/v*) glycerol, and 8 mM β-mercaptoethanol] containing 1 mM phenylmethysulfonyl fluoride, and then homogenized in a microfluidizer (Micronox, Korea). The clear lysate was obtained by centrifugation at 35,000 × *g* for 60 min at 4 °C, and then the supernatant was filtered with a 0.45 µm filter to remove cell debris and any aggregated proteins. The filtered supernatants were loaded onto Ni Sepharose High Performance Ni-charged immobilized metal affinity chromatography (IMAC) resin (GE Healthcare, USA) pre-equilibrated with the lysis buffer, and the recombinant proteins were eluted at 80–400 mM imidazole. The eluted protein samples were concentrated using Amicon Ultra-15 Centrifugal Filter Units (Millipore, USA) and then further purified by size-exclusion chromatography with a HiLoad 16/600 Superdex 200 pg column (GE Healthcare) equilibrated with a buffer containing 25 mM Tris–HCl (pH 8.0), 100 mM sodium chloride, and 0.5 mM Tris (2-carboxyethyl) phosphine (TCEP). Purified hMetAP1D protein was concentrated to 25 mg mL^−1^ for crystallization.

### Crystallization, X-ray data collection, and structure determination

The conditions for the crystallization of hMetAP1D were screened by the sitting drop vapor diffusion method in 96-well plates using the Mosquito robot (TTP Labtech, UK) and various commercially available kits (Molecular Dimensions, UK). The crystals were grown at 14 °C with a crystallization solution containing 0.1 M HEPES pH 7.2, 0.1 M carboxylic acids, 17% (*w*/*v*) 2-methyl-2,4-pentanediol (MPD), 17% (*w*/*v*) PEG 1000, and 17% (*w*/*v*) PEG 3350 by mixing equal volumes of the protein at 25 mg mL^−1^. Subsequently, the crystals co-crystallized with or without 5 mM methionine were soaked overnight in the reservoir solution containing 50 mM CoCl_2_. Crystals of diffraction quality were cryoprotected with the reservoir solution containing an additional 5% (*v*/*v*) glycerol and flash-cooled in a nitrogen gas stream at 100 K. Diffraction data from the apo, Co-bound, and Co-Met-bound forms were collected at resolutions of 1.38, 1.49, and 1.45 Å, respectively. The raw X-ray diffraction data were indexed and scaled using the XDS program package^35^. For the structure of the hMetAP1D-apo form, molecular replacement was performed using Phaser in the PHENIX software suite^36^ with the hMetAP1 structure (PDB code: 2B3K) as the search model. The initial model was further refined to the final model using iterative cycles of model building with Coot^37^ and subsequent refinement with Refmac5 in the CCP4 suite^38^ and phenix.refine^39^. The crystal structures of the Co-bound and Co-Met-bound forms were determined by molecular replacement with the MolRep program^40^, using the refined structure of the unbound form as the phasing model. Validation of crystal structures was implemented using MolProbity^41^ and the Research Collaboratory for Structural Bioinformatics (RCSB) PDB validation server. The statistics for the data collection and refinement are summarized in Table 1.

### Sequence alignment of MetAPs

Structure-based multiple sequence alignment of hMetAP1D(UniProt ID: Q6UB82; PDB code: 8KHM), hMetAP1 (UniProt: P53582; PDB: 2B3K), and hMetAP2 (UniProt: P50579; PDB: 1B6A), ecMetAP (UniProt: P0AE18; PDB:1C21), and mtMetAP (UniProt: P9WK19; PDB: 3IU7) were performed using the PROMALS3D program^42^. The alignment was displayed using ESPript 3.0 software^43^.

### Molecular docking simulation

Docking studies were conducted using Maestro Molecular Docking Simulation of Schrödinger. Receptor and ligand-based grid was generated for docking with the SP docking protocol. This was performed using the Grid-based Ligand Docking with Energetics (Glide) module of Schrödinger version 2.4. All docking runs were performed after removing ligands and water molecules, with the exception of cobalt ions, from the Co-Met-bound hMetAP1D structure. The protein preparation was further energy minimized to refine steric clashes with the restrained minimization of the Optimized Potentials for Liquid Simulations (OPLS)_2005 force field. Coordinates for the dipeptides (Met-Pro or Met-Thr) and tripeptide (Met-Pro-Gln) were generated as ligands using the PyMol builder tool (PyMOL Molecular Graphics System, DeLano Scientific, Palo Alto, USA) and were further prepared using the LigPrep module. The bond orders were fixed, and all the ionization states for the ligand structures were specified at the certain pH of 7.0 ± 2 using Epik. Using the OPLS 2005 force field, a maximum of 32 possible conformations at a given pH were generated for each ligand. The docking process involved prediction of the conformation and orientation (pose) of the ligand within the binding site of the target molecule. Poses with the best docking scores for each ligand were selected for further interaction analyses. These top poses were further confirmed based on the lowest MMGBSA binding free energy (ΔG_bind_), which was calculated via the MMGBSA module in Schrödinger suite 2021-2, under the variable-dielectric generalized born (VSGB) solvation model and the OPLS_2005 force field.

## Data Availability

The coordinates and structure factors of apo-, Co-, and Co-Met-bound hMetAP1D have been deposited in the PDB (http://www.rcsb.org) under accession codes 8KHM, 8KHN, and 8KHO, respectively. Materials supporting the findings of this study are available from the corresponding authors upon request.
